# Does Vitamin D Work Synergistically with Anti-Asthmatic Drugs in Airway Remodeling?

**DOI:** 10.3390/ijms232112798

**Published:** 2022-10-24

**Authors:** Marharyta Sobczak, Rafał Pawliczak

**Affiliations:** Department of Immunopathology, Division of Biomedical Science, Faculty of Medicine, Medical University of Lodz, 90-752 Lodz, Poland

**Keywords:** vitamin D, calcitriol, 1,25(OH)_2_D_3_, bronchial asthma, airway remodeling

## Abstract

Vitamin D is commonly known for its properties of airway remodeling inhibition. Due to this, we decided to analyze the action of calcitriol with anti-asthmatic drugs in airway remodeling. The HFL1 cell line was treated with calcitriol, beclomethasone 17-propionate, montelukast sodium, LTD4 and TGF-β in different combinations. Real-time PCR was used to analyzed the expression of ACTA2, CDH-1, Vimentin, ADAM33, MMP-9 and CysLTR1 on the mRNA level, whereas Western blot was used to analyze gene expression on the protein level. One-way analysis variants, the Kruskal-Wallis test, Student’s *t*-test or Welch’s *t*-test were used for statistical analysis. Concerning the results, pre-treatment with calcitriol increased the inhibitory effect of beclomethasone 17-propionate and montelukast sodium on the expression of *ACTA2* (*p* = 0.0072), *Vimentin* (*p* = 0.0002) and *CysLTR1* (*p* = 0.0204), and 1,25(OH)_2_D_3_ had an influence on the effects of beclomethasone 17-propionate and montelukast sodium and of *CDH1* expression (*p* = 0.0076). On the protein level, pre-treatment with calcitriol with beclomethasone 17-propionate and montelukast sodium treatment decreased ACTA2 expression in comparison to the LT (LTD4 and TGF-β) control group (*p* = 0.0191). Hence, our study not only confirms that vitamin D may inhibit airway remodeling, but also shows that vitamin D has a synergistic effect with anti-asthmatic drugs.

## 1. Introduction

Bronchial asthma is one of the most common inflammatory lung diseases, currently affecting approximately 1–18% population worldwide [1,2]. It causes respiratory symptoms which, in some cases, require intensive care and may be fatal [3]. Rapid symptom control as a result of proper and early diagnosis of asthma is the primary goal of treatment, leading to a reduction in the risk of exacerbations and progressive loss of lung function [4]. 

However, patients often have problems that are associated with corticosteroid therapy. One of the problems observed in children treated with those drugs is growth deficiency. Daily intake of systemic corticosteroids has been shown to suppress growth rate until treatment is discontinued irrespective of the dose, whereas in case of inhaled corticosteroids, the effect depends on the dose, schedule and method of administration. Moreover, growth inhibition caused by systemic corticosteroids can be minimized by discontinuation of therapy before adolescence [5]. An important problem related to steroids is also resistance to steroids in severe asthma, which may result from the incorrect diagnosis of patients, as well as psychological problems and non-compliance with medical recommendations by patients [6]. 

Myofibroblasts are involved in pathological and physiological conditions, thus they secrete extracellular matrix proteins in diseases related to fibrosis. In the course of fibrosis, there is a local infiltration of fibroblasts, which may differentiate into myofibroblasts (mainly in the skin). In the lungs, on the other hand, myofibroblasts can originate in another mechanism—the epithelial-mesenchymal transition (EMT). Moreover, TGF-β1 was considered a powerful regulator of the myofibroblast phenotype, as it can stimulate the transition of fibroblasts to myofibroblasts [7]. Under normal circumstances, fibroblasts, which are αSMA negative, produce small amounts of extracellular matrix and have poor intercellular junctions. However, after tissue damage, fibroblasts are activated and migrate to the site of damage and produce extracellular matrix components. A mechanical change in the microenvironment may also lead to a phenotype change, as the cross-linked extracellular matrix that protects fibroblasts against stress is lost. As a result, protomyofibroblasts are formed, which are an intermediate stage in the formation of differentiated myofibroblasts and contain contractile stress fibers linked to extracellular matrix proteins. Differentiated myofibroblasts are characterized by the expression of α-SMA in stress fibers, which results in increased contractile activity of these cells [8]. In human primary airway epithelial cells, TGF-β1 induced Smad3-dependent EMT accompanied by expression of α-SMA and vimentin with subsequent loss of E-cadherin and zonular occludin-1 expression [9]. Metalloproteinases also play a crucial role in airway remodeling. For example, the expression of a disintegrin and metalloproteinase-33 (ADAM33), which belongs to the ADAM metalloproteinase family, is associated with airway inflammation, hypersensitivity and remodeling in asthmatic patients. Moreover, the silencing of this gene led to proliferation inhibition and induced the apoptosis of human aortic smooth muscle cells [10]. Matrix metalloproteinases (MMPs) are important in extracellular matrix degradation. The major theory of airway remodeling progression involves the disruption of balance in tissue inhibitors of metalloproteinase 1 (TIMP-1) and MMP-9 levels [11]. 

Vitamin D is a member of the group of fat-soluble vitamins. Most vitamin D originates from the skin via synthesis caused by ultraviolet B radiation. In addition to this synthesis, a small amount of the vitamin can be obtained from foods such as: fatty fish, liver or egg yolks. Synthesized vitamin D3 is biologically inactive and must undergo further transformation to its active form—calcitriol [12]. It has been shown that low vitamin D levels can increase the risk of respiratory infections and asthma [13]. A meta-analysis conducted on 25 randomized clinical trials showed that supplementation reduces the risk of acute respiratory infection [14]. Moreover, calcitriol inhibited the ERK signaling pathway and calpain-1 expression, which was induced by cigarette smoke extracts [15]. Moreover, unlike numerous in vitro and in vivo studies, which indicate that vitamin D may positively influence the characteristic features of a given disease, clinical studies show conflicting results [16]. Therefore, we would like to analyze the effect of vitamin D on airway remodeling and the action with anti-asthmatic drugs, such as beclomethasone 17-propionate and montelukast sodium.

## 2. Results

### 2.1. The Influence of Calcitriol on ACTA2 Expression Depends on Dose and Duration of Treatment

**Figure 1:**
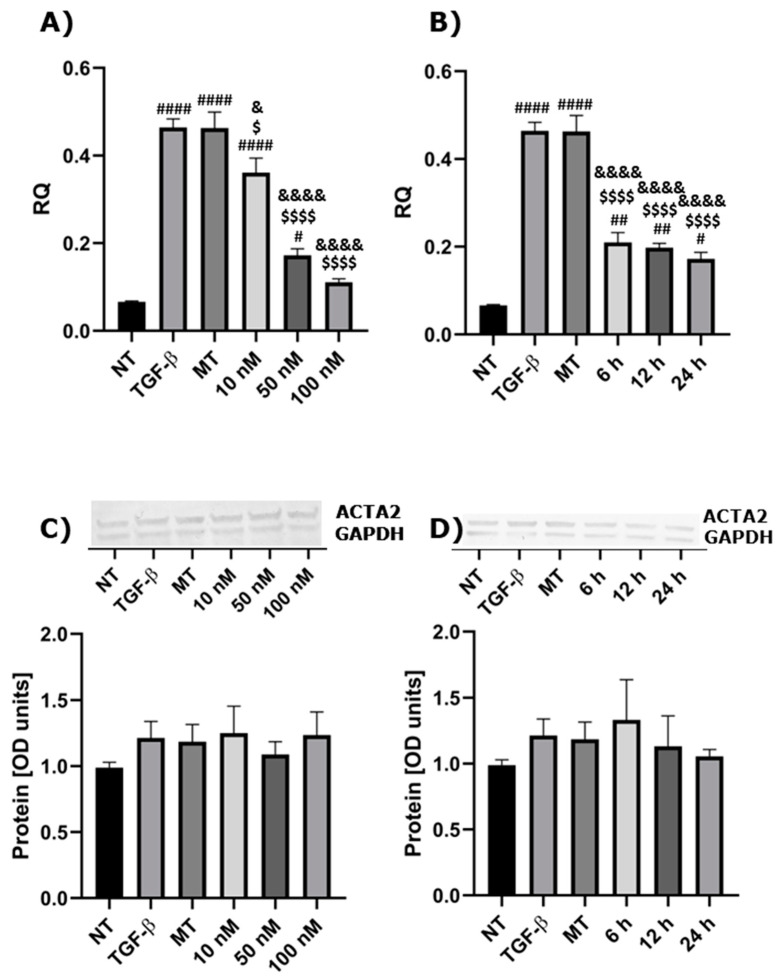
Calcitriol inhibits the expression of ACTA2 in dose- or time-dependent manner. (**A**) mRNA expression and (**C**) protein level of ACTA2 after pre-treatments with three chosen concentrations of 1,25(OH)_2_D_3_ and treatment with 10 ng/mL of TGF-β. (**B**) mRNA expression and (**D**) protein level of ACTA2 after pre-treatments with 1,25(OH)_2_D_3_ for three chosen incubation times and treated with 10 ng/mL of TGF-β. The results of experiments (n = 3) are presented in relation to GAPDH as a mean ± SEM. Western blots are shown for genes, in which the statistical significance has been observed at the mRNA level. Original Western blots can be found at Figure S1. # *p* < 0.05, ## *p* < 0.01, #### *p* < 0.0001 versus NT group; $ *p* < 0.05, $$$$ *p* < 0.0001 versus TGF-β group; & *p* < 0.05 and &&&& *p* < 0.0001 versus MT group. One-way analysis of variance (ANOVA) or Kruskal-Wallis test followed by corresponding post hoc was used to determine differences in several groups, and Student’s *t*-test or Welch’s *t*-test was used to determine statistically significant differences between two means. RQ—relative quantification of genes expression normalized to GAPDH, NT—non-treatment cells, TGF-β—cells treated with TGF-β, MT—cells treated with 0.1% of methanol and TGF-β.

Firstly, we aimed to identify the appropriate treatment conditions. For this, we assessed the inhibitory capability of three concentrations of 1,25(OH)_2_D_3_ on the expression of ACTA2, the marker of myofibroblasts. The expression of *ACTA2* was significantly decreased after treatment with all analyzed concentrations of 1,25(OH)_2_D_3_ on mRNA levels compared to control groups, similarly for TGF-βand MT (cells treated with 0.1% of methanol and TGF-β) (Figure 1A). However, regarding protein levels, the expression was similar among groups (Figure 1C). Moreover, we checked three different incubation times. During all analyzed incubation periods (6 h, 12 h and 24 h), the expression of *ACTA2* was significantly decreased on the mRNA level (Figure 1B), but without being statistically significant on protein levels (Figure 1D). For subsequent experiments, we chose the concentration of 100 nM of 1,25(OH)_2_D_3_ and an incubation time of 24 h.

### 2.2. The Influence of Calcitriol, Beclomethasone 17-Propionate and Montelukast Sodium on ACTA2, CDH1 and Vimentin Expression

**Figure 2:**
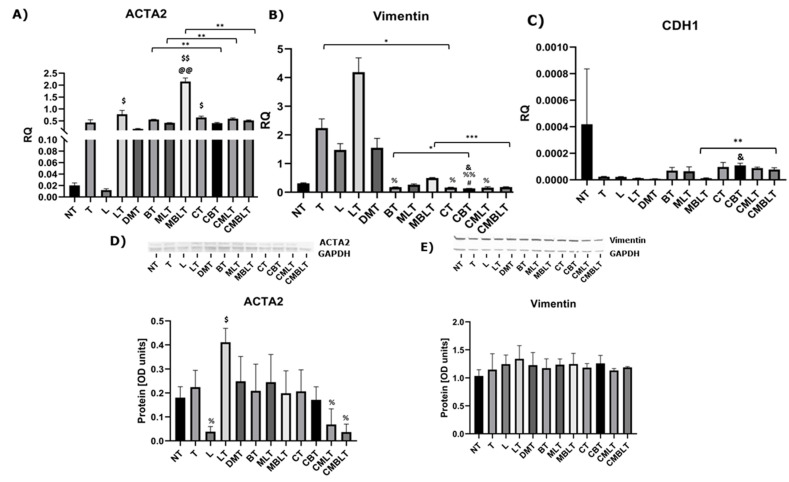
Calcitriol, beclomethasone 17-propionate and montelukast sodium effect on the expression of ACTA2, CDH1 and Vimentin. (**A**–**C**) mRNA expression and (**D**,**E**) protein level of ACTA2, CDH-1 and Vimentin after exposure to calcitriol, beclomethasone 17-propionate, montelukast sodium, LTD4 and TGF-β. The results of experiments (n = 3) are presented in relation to GAPDH as mean ± SEM. Western blots are shown for genes, in which the statistical significance has been observed at the mRNA level. Original Western blots can be found in Figure S1. @@ *p* < 0.01 versus NT group; # *p* < 0.05 versus T group; $ *p* < 0.05, $$ *p* < 0.01 versus L group; % *p* < 0.05, %% *p* < 0.01 versus LT group; and & *p* < 0.05 versus DMT group; * *p* < 0.05, ** *p* < 0.01, *** *p* < 0.001. One-way analysis of variance (ANOVA) or Kruskal-Wallis test followed by corresponding post hoc was used to determine differences in several groups, and Student’s *t*-test or Welch’s *t*-test was used to determine statistically significant differences between two means. RQ—relative quantification of genes expression normalized to GAPDH, NT—untreated cells, T—cells treated with TGF-β, L—cells treated with LTD4, LT—cells treated with LTD4 and TGF-β, DMT—cells treated with methanol, DMSO and TGF-β, BT—cells treated with beclomethasone 17-propionate and TGF-β, MLT—cells treated with montelukast sodium, LTD4 and TGF-β, MBLT—cells treated with beclomethasone 17-propionate, montelukast sodium, LTD4 and TGF-β, CT—cells treated with calcitriol and TGF-β, CBT—cells treated with calcitriol, beclomethasone 17-propionate and TGF-β, CMLT—cells treated with calcitriol, montelukast sodium, LTD4 and TGF-β, CBMLT—cells treated with calcitriol, beclomethasone 17-propionate, montelukast sodium, LTD4 and TGF-β.

We analyzed the expression of ACTA2, Cadherin-1 (CDH1) and Vimentin as EMT markers (Figure 2A–E). Interestingly, the expression of *ACTA2* was increased after treatment with beclomethasone 17-propionate, montelukast sodium, LTD4 and TGF-β versus NT (non-treated cells) group (*p* = 0.0046) and L (cells treated with LTD4) group (*p* = 0.0016). This effect was decreased after pre-treatment with calcitriol (*p* = 0.0072). Regarding protein levels, pre-treatment with calcitriol with beclomethasone 17-propionate, montelukast sodium, LTD4 and TGF-β treatment also decreased the ACTA2 expression in comparison to the LT control group (cells treated with LTD4 and TGF-β) (*p* = 0.0191). *Vimentin* expression was decreased in BT (cells treated with beclomethasone 17-propionate and TGF-β), CT (cells treated with calcitriol and TGF-β), CBT (cells treated with calcitriol, beclomethasone 17-propionate and TGF-β) and CMLT (cells treated with calcitriol, montelukast sodium, LTD4 and TGF-β) groups in comparison to the LT group (cells treated with LTD4 and TGF-β). Moreover, calcitriol significantly decreased the *Vimentin* expression compared to TGF-β stimulation (*p* = 0.0229), as well as increased the inhibitory effects of beclomethasone 17-propionate (*p* = 0.0323), and cumulative effects of beclomethasone 17-propionate and montelukast sodium (*p* = 0.0002). Similarly, 1,25(OH)_2_D_3_ had a synergistic influence on the effects of beclomethasone 17-propionate and montelukast sodium on increasing *CDH1* expression (*p* = 0.0076). 

### 2.3. The Influence of Calcitriol, Beclomethasone 17-Propionate and Montelukast Sodium on Expression of Metalloproteinases MMP-9 and ADAM33

**Figure 3:**
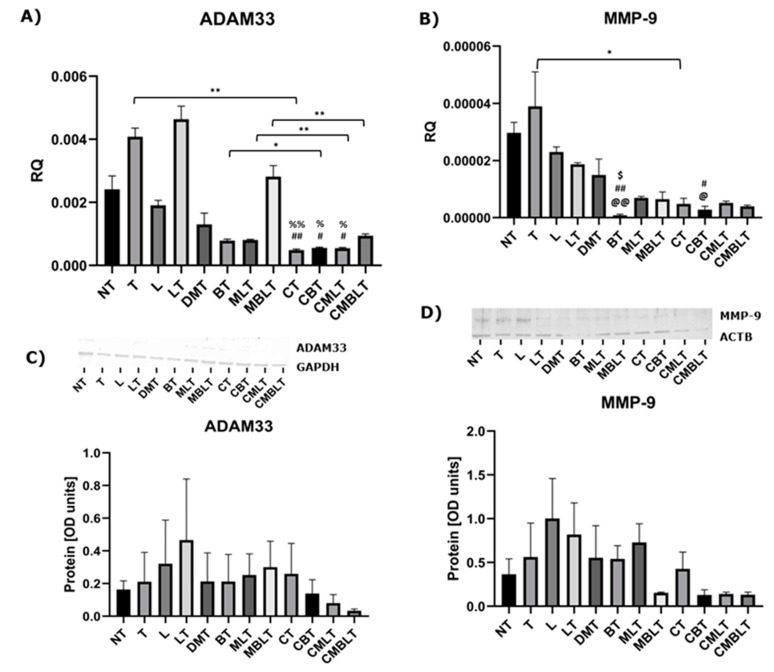
Calcitriol, beclomethasone 17-propionate and montelukast sodium effect on the expression of Vimentin, ADAM33 and MMP-9. (**A**,**B**) mRNA expression and (**C**,**D**) protein level of ADAM33 and MMP-9 after exposure to calcitriol, beclomethasone 17-propionate, montelukast sodium, LTD4 and TGF-β. The results of experiments (n = 3) are presented in relation to GAPDH or ACTB as mean ± SEM. Western blots are shown for genes, in which the statistical significance has been observed at the mRNA level. Original Western blots can be found at Figure S1. @ *p* < 0.05, @@ *p* < 0.01 versus NT group; # *p* < 0.05, ## *p* < 0.01 versus T group; $ *p* < 0.05 versus L group; % *p* < 0.05, %% *p* < 0.01 versus LT group; * *p* < 0.05, ** *p* < 0.01. One-way analysis of variance (ANOVA) or Kruskal-Wallis test followed by corresponding post hoc was used to determine differences in several groups, and Student’s *t*-test or Welch’s *t*-test was used to determine statistically significant differences between two means. RQ—relative quantification of genes expression normalized to GAPDH, NT—untreated cells, T—cells treated with TGF-β, L—cells treated with LTD4, LT—cells treated with LTD4 and TGF-β, DMT—cells treated with methanol, DMSO and TGF-β, BT—cells treated with beclomethasone 17-propionate and TGF-β, MLT—cells treated with montelukast sodium, LTD4 and TGF-β, MBLT—cells treated with beclomethasone 17-propionate, montelukast sodium, LTD4 and TGF-β, CT—cells treated with calcitriol and TGF-β, CBT—cells treated with calcitriol, beclomethasone 17-propionate and TGF-β, CMLT—cells treated with calcitriol, montelukast sodium, LTD4 and TGF-β, CBMLT—cells treated with calcitriol, beclomethasone 17-propionate, montelukast sodium, LTD4 and TGF-β.

We also checked the effect of calcitriol and analyzed drugs on the expression of metalloproteinase MMP-9 and ADAM33 on mRNA and protein levels, which is shown in Figure 3 (A–D). The expression of *ADAM33* was significantly decreased in CT (cells treated with calcitriol and TGF-β), CBT (cells treated with calcitriol, beclomethasone 17-propionate and TGF-β) and CMLT (cells treated with calcitriol, montelukast sodium, LTD4 and TGF-β) groups compared to T (cells treated with TGF-β) and LT (cells treated with LTD4 and TGF-β) control groups. Similarly, *MMP-9* expression was also significantly decreased in BT and CBT groups. Calcitriol significantly decreased the expression of *ADAM33* and *MMP-9* in comparison to TGF-β stimulation (*p* = 0.0051 and *p* = 0.0500, respectively). Moreover, pre-treatment with 1,25(OH)_2_D_3_ enhanced *ADAM33* expression inhibition after beclomethasone 17-propionate treatment (*p* = 0.0129), montelukast sodium treatment (*p* = 0.0024), as well as on beclomethasone 17-propionate and montelukast sodium treatment (*p* = 0.0062). However, the inhibitory effect was not present on protein levels.

### 2.4. The Influence of Calcitriol, Beclomethasone 17-Propionate and Montelukast Sodium on CysLTR1 Expression

**Figure 4:**
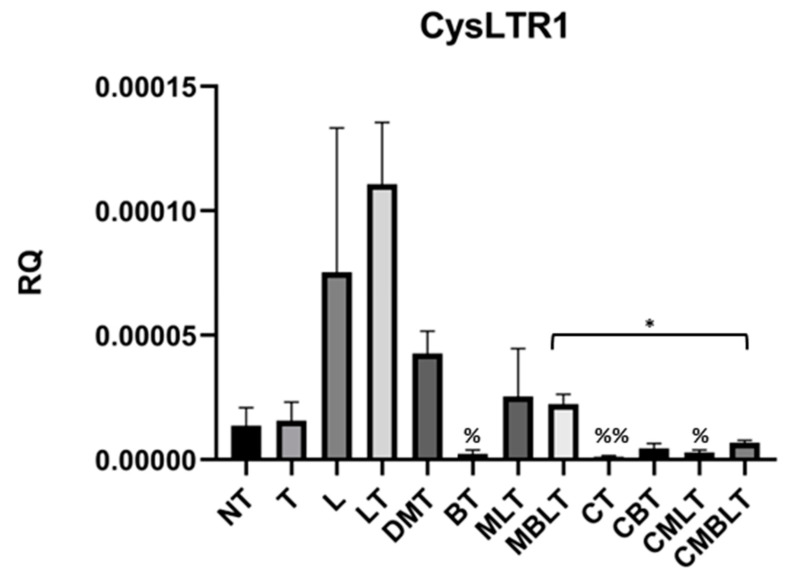
Calcitriol, beclomethasone 17-propionate and montelukast sodium effect on the expression of CysLTR1. mRNA expression CysLTR1 after exposure to calcitriol, beclomethasone 17-propionate, montelukast sodium, LTD4 and TGF-β. The results of experiments (n = 3) are presented in relation to GAPDH as mean ± SEM. Western blots are shown for genes, in which the statistical significance has been observed at the mRNA level. Original Western blots can be found at Figure S1. % *p* < 0.05, %% *p* < 0.01 versus LT group; * *p* < 0.05. One-way analysis of variance (ANOVA) or Kruskal-Wallis test followed by corresponding post hoc was used to determine differences in several groups, and Student’s *t*-test or Welch’s *t*-test was used to determine statistically significant differences between two means. RQ—relative quantification of genes expression normalized to GAPDH, NT—untreated cells, T—cells treated with TGF-β, L—cells treated with LTD4, LT—cells treated with LTD4 and TGF-β, DMT—cells treated with methanol, DMSO and TGF-β, BT—cells treated with beclomethasone 17-propionate and TGF-β, MLT—cells treated with montelukast sodium, LTD4 and TGF-β, MBLT—cells treated with beclomethasone 17-propionate, montelukast sodium, LTD4 and TGF-β, CT—cells treated with calcitriol and TGF-β, CBT—cells treated with calcitriol, beclomethasone 17-propionate and TGF-β, CMLT—cells treated with calcitriol, montelukast sodium, LTD4 and TGF-β, CBMLT—cells treated with calcitriol, beclomethasone 17-propionate, montelukast sodium, LTD4 and TGF-β.

We checked if calcitriol, beclomethasone 17-propionate and montelukast sodium administration affect CysLTR1 expression. Figure 4 shows that TGF-β did not increase the expression of CysLTR1, although the expression was increased after treatment with LTD4 and TGF-β (LT group) (*p* > 0.05). Expression of this gene was significantly decreased after treatment with beclomethasone 17-propionate (*p* = 0.0143), calcitriol (*p* = 0.0082) or calcitriol and beclomethasone 17-propionate (*p* = 0.0213) compared to control (LT). Moreover, calcitriol pre-treatment significantly strengthened the inhibition of *CysLTR1* expression after beclomethasone 17-propionate and montelukast sodium treatment (*p* = 0.0204).

## 3. Discussion

In our study, we aimed to assess the anti-airway remodeling efficacy of vitamin D in combination with commonly prescribed anti-asthmatic drugs such as beclomethasone 17-propionate and montelukast sodium. Although calcitriol and beclomethasone 17-propionate single-treatments led to a substantial decrease of pro-remodeling phenotype traits acquired after TGF-1β or TGF-1β/LTD4 administration, we observed no effect of montelukast sodium even on mRNA levels. However, we observed the synergistic effect of combined treatments in HFL1 cells. Calcitriol may increase the cumulative effect of beclomethasone 17-propionate and montelukast sodium on airway remodeling, but mainly on mRNA levels. 

Positive outcomes of calcitriol administration in asthma are well known and have been confirmed in multiple papers to date. In pediatric patients with asthma, lower calcitriol levels in serum were related to more severe asthma [17]. Interestingly, adult asthmatic vitamin D-deficient patients were characterized by worsened lung function and impaired therapy response [18]. In elderly asthmatic patients, vitamin D insufficiency or deficiency is relatively common, and 12-week supplementation of calcitriol improved asthma control test results [19]. According to the literature, calcitriol is a relatively potent inhibitor of TGF-β signaling. Vitamin D attenuated the expression of particular EMT predictors such as Vimentin, E-cadherin, MMP-2 or MMP-9 in the epithelium model BEAS-2B cell line [20]. Huang et al. [21] acknowledged the lowered level of vitamin D in the serum of asthmatic rats. Moreover, vitamin D administration reduced the expression of particular pro-remodeling factors such as Wnt5a, ACTA2 or β-catenin. In airway epithelial cells, the 1,25(OH)_2_D_3_ limited the changes in epithelium done by cigarette smoke extract. It reduced the expression of calpain-1 as well as ERK phosphorylation, thus hampering cleavage of E-cadherin and pathological epithelial permeability [15]. The abovementioned examples are in line with our results, as vitamin D alone decreased expression of particular EMT markers such as Vimentin. Moreover, Song et al. [22] reported that calcitriol inhibited the expression of metalloproteinases such as ADAM33 and MMP-9 in human airway smooth muscle cells after asthma serum stimulations. In other studies, the inhibition effect of ADAM33 [23] and MMP-9 [24] expression by calcitriol was also observed. Importantly, in our study, we observed a similar effect on mRNA levels. 

The potential synergistic effect of the beclomethasone and calcitriol model of asthma is not well-described in the literature so far. However, the synergistic effect between calcitriol and another member of the glucorticosteroid family, budesonide, is fairly well described. In the study by Xu et al. [25], a cumulative effect of budesonide and calcitriol has been presented. TGF-β1 stimulates airway remodeling via the TGF-β/Smad signaling pathway and regulation of miR-21 expression. Calcitriol inhibited the pathway and miR-21 expression, thus hampering airway remodeling. The effect was enhanced by budesonide treatment. According to the described mechanism, both calcitriol and budesonide induced the expression of the vitamin D receptor, although, when introduced simultaneously, the effect was significantly stronger. Similar conclusions were drawn in the study by Qian et al. [26], where the abovementioned interaction had been tested in vivo in a mouse asthma model. The combination treatment improved airway remodeling in mice in a similar fashion by increasing the expression of VDR. Moreover, lower levels of vitamin D corresponded to worse lung function in asthmatic children treated with budesonide [27]. Considering the abovementioned examples of synergy between budesonide and calcitriol, we decided to test the effect of beclomethasone17-propionate. In our study, although we observed the high anti-remodeling potency of beclomethasone 17-propionate and vitamin D separately, we also observed a synergistic effect between them. 

Similarly to beclomethasone, the effect of combination of montelukast and calcitriol remains poorly described. In a murine asthma model, introduction of montelukast prevented airway remodeling [28]. Moreover, pre-treatment with montelukast inhibited the morphological changes in human lung epithelial cells (BEAS-2b), which were induced by eosinophils. Those changes included increased the E-cadherin expression and decreased Collagen I and Vimentin expression [29]. However, in our study, stimulation by montelukast did not significantly affect analyzed markers. Interestingly, in murine models, montelukast, unlike dexamethasone, decreased airway hyperresponsiveness [30], although, it is well-known that the combination of inhaled glucocorticoids and montelukast was proven to be beneficial in asthmatic patients [31]. However, we observed enhancement of ACTA2 expression following combined treatment with beclomethasone 17-propionate and montelukast sodium. Moreover, montelukast sodium as well as montelukast sodium with beclomethasone 17-propionate did not decrease CysLTR1 expression, although, pre-treatment with calcitriol led to inhibition of CysLTR1 expression. Chibana et al. [32] showed that without stimulation, HFL-1 cells have low CysLTR1 levels, unlike human alveolar macrophages and human bronchial smooth muscle cells, but the mRNA expression was stimulated following treatment with IL-13. 

Clinical studies about supplementation of vitamin D in bronchial asthma show inconsistent results. On the one hand, several studies presented no effect of vitamin D supplementation on improving time to a severe as well as a virally induced severe exacerbation and reduction of the dose of inhaled corticosteroid [33], on time to the first severe exacerbation [34] and the exacerbation risk [35]. On the other hand, vitamin D supplementation improved asthma control levels based on the Global Initiative for Asthma in Japanese school children [36], improved forced expiratory volume in 1 second (FEV1) in mild and moderate persistent asthma [37], decreased the number of asthma exacerbations and the steroids requirement in children [38]. Despite the data from clinical trials, our study not only confirms the important role of vitamin D in inhibition airway remodeling, but also shows that vitamin D may improve the anti-remodeling properties of anti-asthmatic drugs on mRNA levels.

## 4. Materials and Methods

### 4.1. Materials

The HFL1 cell line was purchased from ATCC (Manassas, VA, USA). HAM’s12 medium, L-glutamine, Penicillin-Streptomycin solution, 1,25(OH)_2_D_3_, Montelukast sodium, Leukotriene D4, goat anti-rabbit IGG, goat anti-mouse IGG, protease inhibitor cocktail, RIPA buffer and CYSLTR1 antibody were purchased from Merck (Darmstadt, Germany). Fetal bovine serum was purchased from Genos (Lodz, Poland). Beclomethasone 17-Propionate and primary antibodies such as E-cadherin (sc-8426), MMP-9 (sc-393859), ADAM33 (sc-514055), α-Actin (sc-32251), GAPDH (sc-47724) and Vimentin (sc-6260) were purchased from Santa Cruz Biotechnology, Inc. (Dallas, TX, USA). Non-essential amino acids solution, TGFB1 Recombinant Human Protein, TRIzolTM Plus RNA Purification Kit, High-Capacity cDNA Reverse Transcription Kit, TaqManTM Gene Expression Master Mix, TaqMan™ gene expression assays: ACTA2 (Hs00426835_g1), MMP-9 (Hs009557562_m1), VIM (Hs00958111_m1), CysLTR1 (Hs00272624_s1), ADAM33 (Hs00905552_m1), CDH1 (Hs01023895_m1) and GAPDH (Hs02786624_g1) were purchased from Thermo Fisher Scientific (Waltham, MA, USA). Lastly, 4–20% ExpressPlus PAGE gel and MOPS Running buffer powder were purchased form GenSignal (Poznan, Poland).

### 4.2. Cell Cultures and Treatments

HFL1 cells, which are normal, are mortal human fetal lung fibroblasts. The cellular model has been selected, as fibroblasts cells undergo airway remodeling in asthmatic patients. Cells were cultured in HAM’s12 medium supplemented with 1% non-essential amino acids, 10% FBS, penicillin–streptomycin solution in 5% CO_2_. Then, 24 h before experiments, when the cells reached 80–90% confluence, the medium was changed to free FBS. For the first part, the cells were treated with 10 nM, 50 nM and 100 nM of 1,25(OH)_2_D_3_ for 24 h and 10 ng/mL of TGF-β for next 24 h, as well as with 100 nM of 1,25(OH)_2_D_3_ for 6 h, 12 h and 24 h and 10 ng/mL of TGF-β for next 24 h. For the other experiments, cells were pre-treated with 100 nM of 1,25(OH)_2_D_3_ and/or 1 µM of Beclomethasone 17-Propionate for 24 h and/or 1 µM of Montelukast sodium for 60 min, and, next, treated with and 10 ng/mL of TGF-β and/or 10 nM of LTD4 for 24 h. As control groups, the NT group (cells were treated with medium only), MT or DMT group (cells were treated with 0.1% of methanol or 0.1% of methanol and DMSO with added 10 ng/mL of TGF-β for 24 h), TGF- β or T group (cells were treated 10 ng/mL of TGF-β for 24 h), L group (cells were treated with 10 nM of LTD4 for 24 h) and LT group (cells were treated with 10 ng/mL of TGF-β and 10 nM of LTD4 for 24) were used. Conditions of experiments as well as treatments were based on the literature data, although they were adjusted according to our experimental model. 

### 4.3. Analysis of Gene Expression

Total mRNA was isolated using the TRIzol^TM^ Plus RNA Purification Kit and reverse transcribed using thr High-Capacity cDNA Reverse Transcription Kit according to the producer’s protocols. Gene expression analysis of CDH-1, MMP-9, ADAM33, ACTA2 and Vimentin was assessed using the TaqMan^TM^ Gene Expression Master Mix with TaqMan™ gene expression assays in triplicate according to the producer’s protocols. The 2^−ΔΔCt^ method was used to calculate gene expression. Results were normalized to GAPDH expression. 

### 4.4. Immunoblotting

Total protein was isolated using the RIPA buffer with a protease inhibitor cocktail and separated in 4–20% ExpressPlus PAGE gel for 60 min (110 mA). After this, the protein was transferred into nitrocellulose membrane using the XCell II™ Blot Module. One hour incubation with 5% non-fat milk dissolved in TBST buffer was used to block the membrane. The membrane was incubated with primary antibodies with 1% non-fat milk dissolved in TBST buffer at 4 °C overnight and, next, with secondary antibodies for 90 min at room temperature. The results were visualized using BCIP/NBT alkaline phosphatase substrate. Densitometric image analysis was performed in Image J 1.49 software (Wayne Rasband, National Institutes of Health, Bethesda, Washington, MD, USA). 

### 4.5. Statistical Analysis

The results were analyzed in GraphPad Prism 8 (San Diego, CA, USA). The one-way analysis of variance (ANOVA) or Kruskal-Wallis test followed by corresponding post-hoc was used to determine differences in several groups, and Student’s *t*-test or Welch’s *t*-test was used to determine statistically significant differences between two means. Results were considered statistically significant at *p* < 0.05.

## 5. Conclusions

Despite the controversial results of clinical trials, we suggested that vitamin D supplementation may have a synergistic effect on the treatment of bronchial asthma in the future. This may improve the quality of life of patients with chronic disease, although more research as well as clinical trials are required on this matter.

## Data Availability

The data presented in this study are available in the Supplementary Materials, Figure S1.

## Supplementary Materials

The following supporting information can be downloaded at: www.mdpi.com/article/10.3390/ijms232112798/s1.
