# Supplementary figures and images for: Does Vitamin D Work Synergistically with Anti-Asthmatic Drugs in Airway Remodeling?
Source: Int J Mol Sci. 2022 Oct 24;23(21):12798. doi: 10.3390/ijms232112798 (PMC9656909; doi:10.3390/ijms232112798)

Figure S1

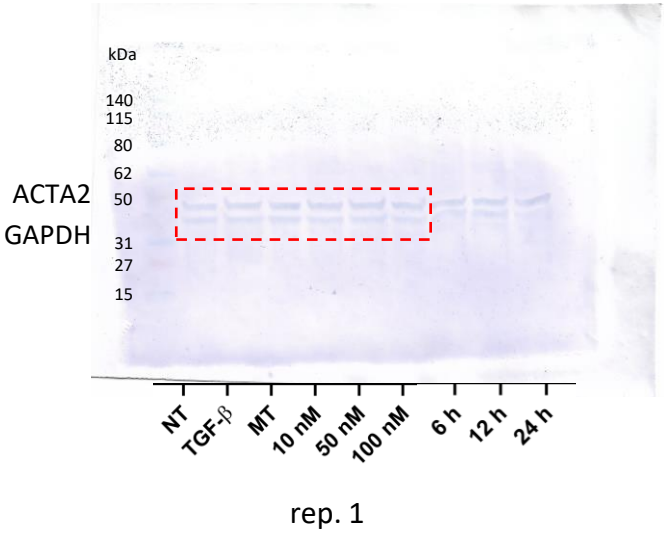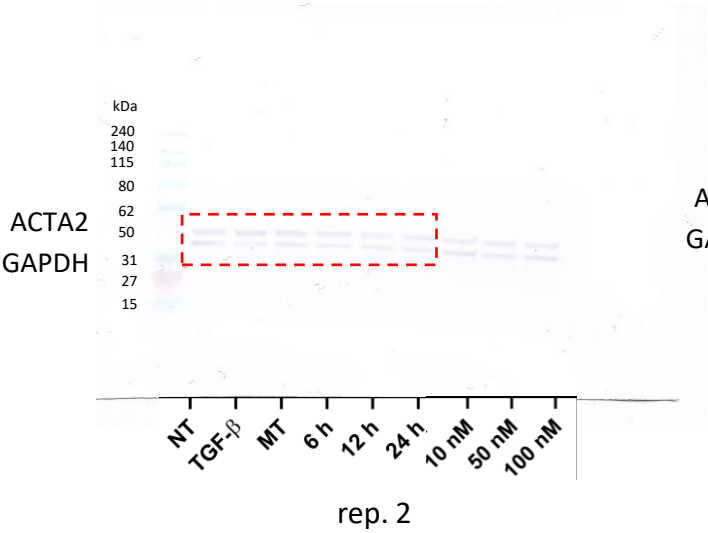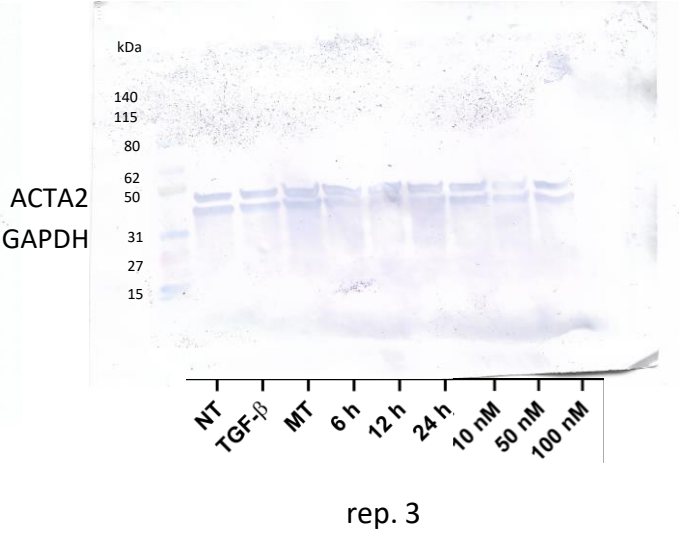

Figure S2

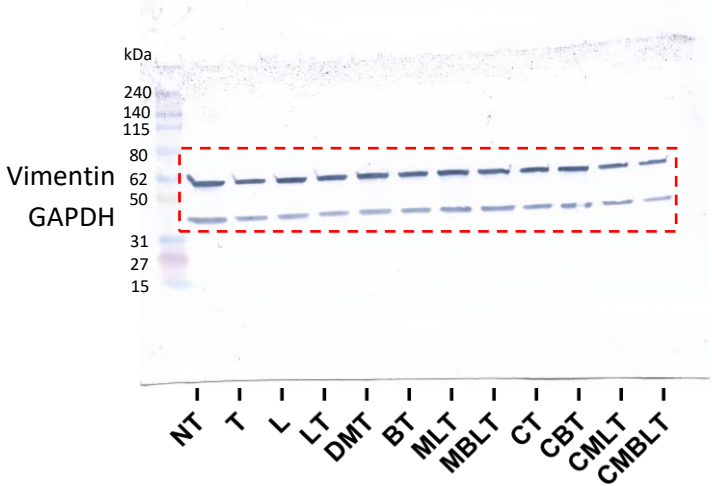

rep. 1

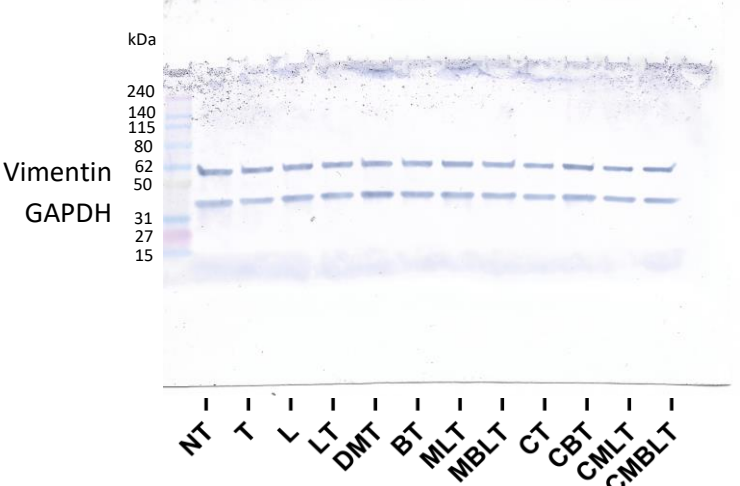

rep. 2

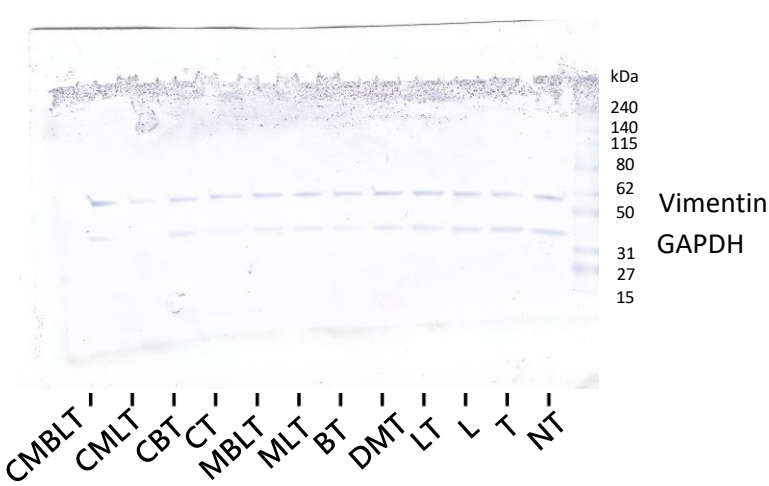

rep. 3

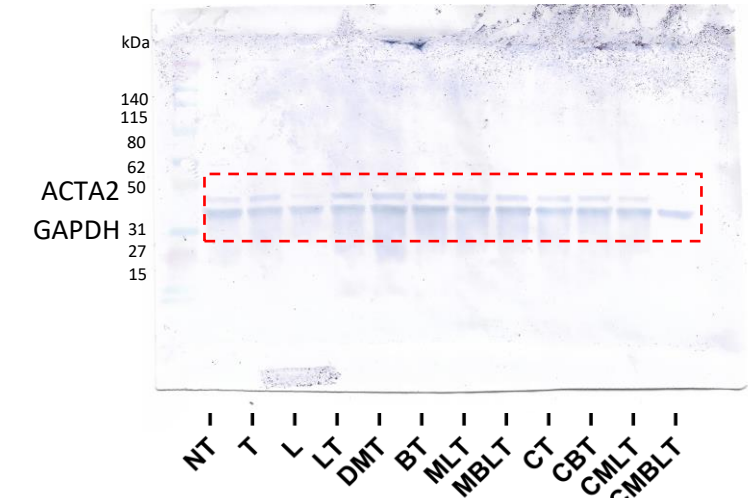

rep. 1

Figure S3

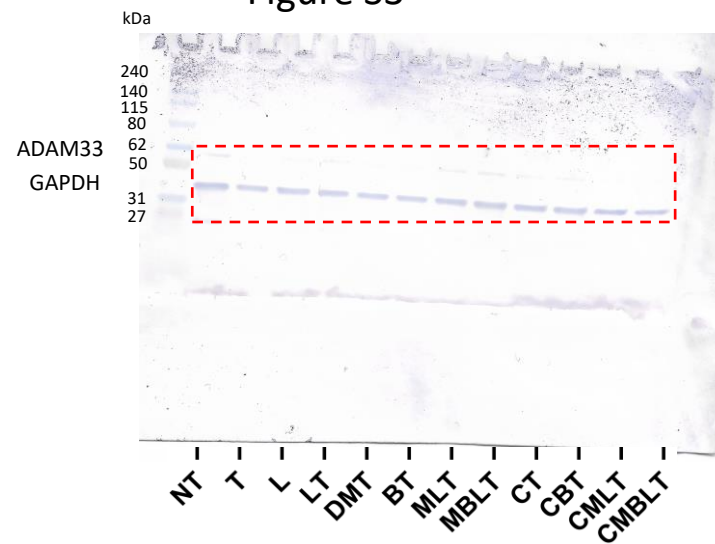

rep. 1

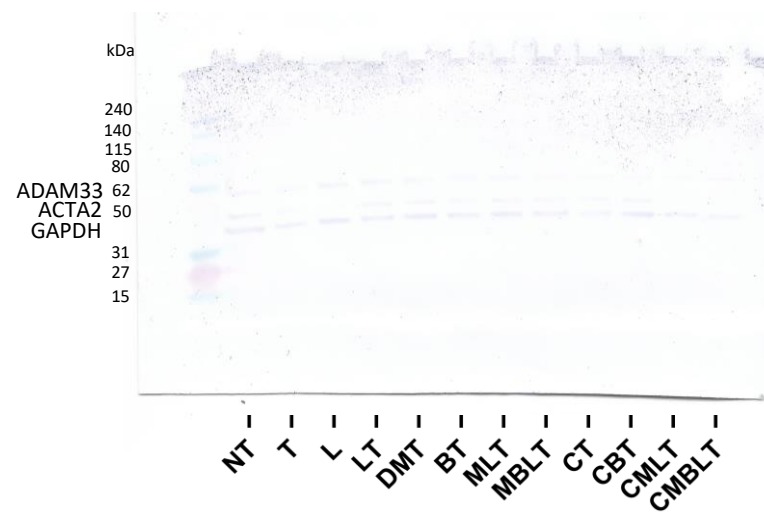

rep. 2

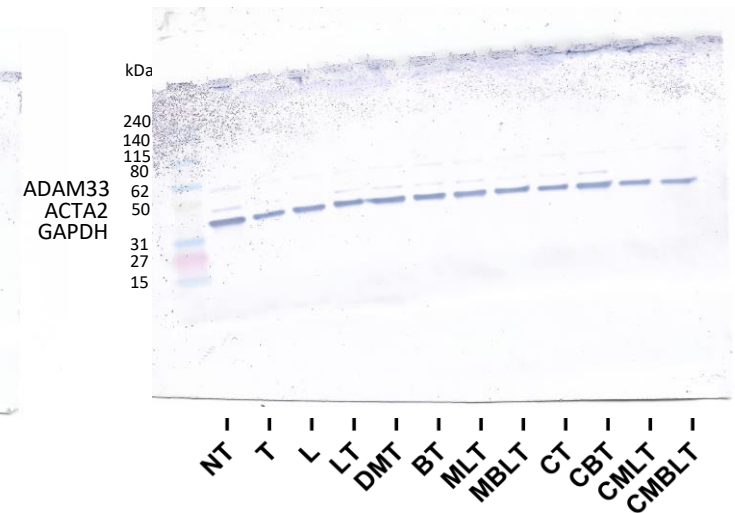

rep. 3

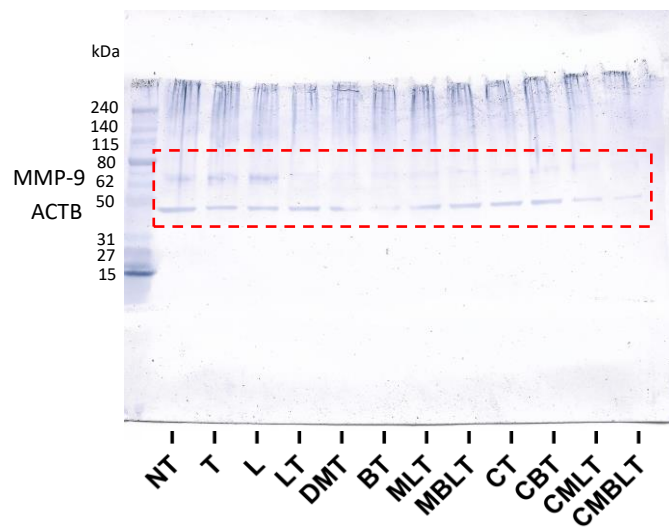

rep. 1

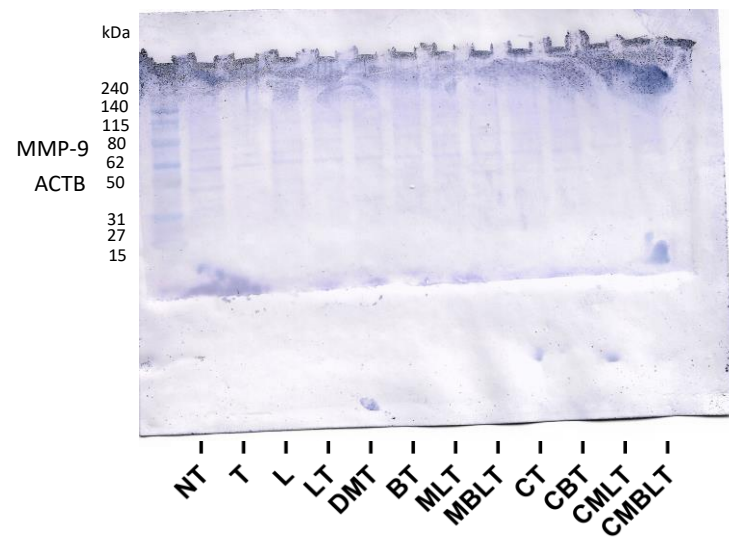

rep. 2

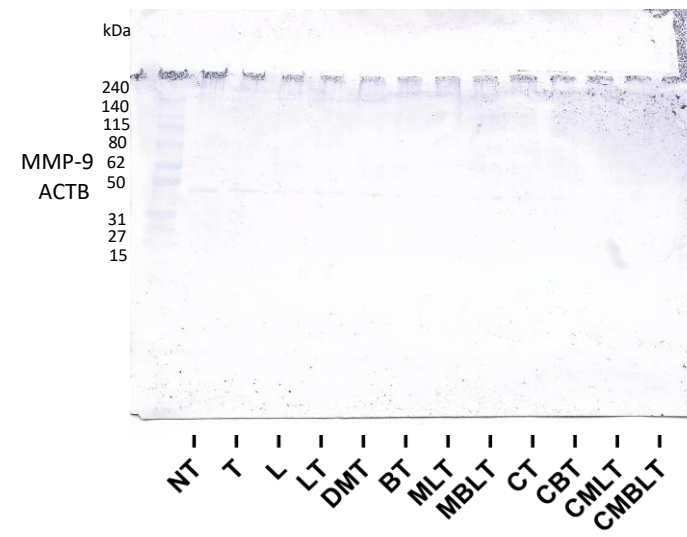

rep. 3

Supplement: Supplementary file 1 [file ijms-23-12798-s001.zip › ijms-1971476-supplementary.pdf]
